# miR-150-5p represses TP53 tumor suppressor gene to promote proliferation of colon adenocarcinoma

**DOI:** 10.1038/s41598-019-43231-5

**Published:** 2019-05-01

**Authors:** Fang Liu, Xiao Di Wang

**Affiliations:** Department of Gastroenterology, China-Japan Friendship Hospital, East Street of Yinghua, Chaoyang District, Beijng, 100029 China

**Keywords:** Cancer microenvironment, Colon cancer

## Abstract

MicroRNAs (miRNAs) play a critical role in regulation of numerous biological processes and pathogenesis of a variety of diseases. In addition, miRNAs contribute to carcinogenesis by acting as oncogenic or tumor suppressive. Circulating miRNAs including miR-150-5p are associated with colorectal cancer progression, and the putative targets of miR-150-5p include tumor suppressor gene, TP53. Here we sought to investigate the role of miR-150-5p-TP53 signaling pathway in proliferation of colon cancer and to determine expression levels of miR-miR-150-5p and TP53 in colon adenocarcinoma and adjacent non-cancerous tissue samples, or in human colon adenocarcinoma cell lines. MTT assay was used to determine proliferation and apoptosis in cell lines. Furthermore, we used Western blot to determine levels of cell cycle regulators with anti-miR-150-5p or apoptosis with overexpression of TP53. Our results show that expression levels of miR-150-5p were significantly elevated in clinical specimens from cancer patients. We further showed that inhibition of miR-150-5p increased TP53, and in turn, suppression of proliferation of colon adenocarcinoma. Moreover, inhibition of miR-150-5p or overexpression of TP53 caused cell arrest or apoptosis in colon adenocarcinoma. Our results support that miR-150-5p-TP53 pathway plays an important role in regulation of proliferation, cell arrest, and apoptosis in colon cancer, and could be an attractive target for therapy.

## Introduction

Colorectal cancer (CRC) is a common malignant tumor with an incidence rate of a million people worldwide every year, and a major cause of mortality and morbidity in developed countries^1–3^. In addition, CRC ranks as the third most common cancer by incidence and the fourth most common cause of cancer-related deaths world-wide. Presently, surgical resection is the only approach for curative treatment of CRC^4^. Despite numerous efforts in the past few decades, CRC survival rates have not significantly improved, and half of the CRCs relapse and the patients die within five years. As such, identifying novel and more effective drug targets in treatment of CRC are imminent.

MicroRNA (miRNAs) usually silence target genes by binding to their target messenger RNAs at 3′-UTR^5^. It has been estimated that miRNAs can regulate one-third of all human genes and influence hundreds of potential gene targets^6^. Dysregulation of miRNA expression is closely linked to cancer initiation and progression. Mechanistically, miRNAs can inhibit oncogenes or tumor suppressor genes to play a tumor suppressor or oncogenic role, respectively^7^. In addition, miRNAs can serve as biomarkers for early cancer diagnosis, because miRNAs are stable in body fluids, either in secreted microvesicles or bound to carrier proteins, which provides a unique opportunity to exploit the role of miRNAs as biomarkers^8^. Indeed, expression levels of circulating miRNAs have shown some potential at differentiating cancer patients and healthy controls for prostate^9^, ovarian^10^, lung^11^ and breast^12^ cancers. Moreover, multiples studies support the prognostic and diagnostic value of some circulating exosomal microRNAs in colon cancer, particularly, miR-150-5p^13,14^.

The tumor suppressor gene, *TP53*, is one of the most frequently mutated genes in many cancers including CRC^15^. TP53 is generally expressed at low levels, in part through negative feedback loops that involve MDM2^16^. Low abundance of T53 expression preserves homoeostasis of the cell cycle and cell death. In response to stress factors, such as hypoxia, UV irradiation, oxidative free radicals, and activation of oncogenes, TP53 protein is activated, and such an activation of p53 can either transactivate or repress downstream target genes that in turn regulate cell cycle arrest, apoptosis, DNA repair, and angiogenesis and even metastasis^17^.

Although multiple studies have revealed the potential tumor-suppressive effect of TP53 in human tumor, the molecular function of TP53 in CRC has remained further defined. Expression of miR-150-5p has been linked with TP53-downstream target genes^15^. Interestingly, a preliminary bioinformatics analysis indicates that TP53 is a potential target of miR-150-5p^18^. In the present study, we sought to investigate the role of miR-150-5p-TP53 signaling in CRC, and we demonstrate that such a signaling plays a critical role in proliferation and progression of CRC.

## Materialas and Methods

### Subjects

We collected specimens from a total of 10 CRC patients after surgical resection. The study and all methods were carried out in accordance with relevant guidelines and regulations. all experimental protocols were approved by the Ethic Committee of China-Japan Friendship Hospital. The general information for patients is provided in Table 1. Informed consent was obtained from all subjects.

**Table 1:** Patient Characteristics.

Age (years)	50–62
Gender Distribution	7 males (70%) and 3 Females (30%)
Histopathological Diagnosis	All Colon Adenocarcinomas
TNM Stage	4 cases (40%) Stage II 6 cases (60%) Stage III

### Cell culture

Normal human colon epithelial cell line CCD 841 CoN (ATCC, USA), and human colon carcinoma cell lines HT29, T84, and LS174 (ATCC, USA) were maintained in RPMI-1640 medium supplemented with 10% fetal bovine serum, as recently described^19^. Cells were cultured in a humidified incubator at 37 °C and 5% CO_2_.

### Reagents

Antibodies to TP53, COX-2, CDK1, Cyclin B1, Caspase-3 and -9 were obtained from Santz Cruz Biotech (TX, USA). TP53 siRNA, miR-150-5p mimics or anti-miR-150-5p inhibitors were purchased from Qiagen (USA).

### Quantitative RT-PCR

Total RNAs were extracted by Trizol method (Invitrogen, CA, USA) and re-suspended in nuclease-treated H_2_O. cDNA synthesis was prepared by the miR-150-5p-, U6 snRNA-specific or oligo-dT primers method using the Superscript II Reverse Transcription kit (Invitrogen, USA). Quantitative PCR was performed using an ABI7300 machine (Applied Biosystems, USA). miR-150-5p or TP53 mRNA levels were determined, relative to U6 or GAPDH expression using the SYBR Green PCR kit (Qiagen, USA), respectively. Fold change in expression was determined by the method of cycle threshold (CT) using the formula of 2^−ΔΔCT^. U6 forward, 5′-TGCGGGTGCTCGCTTCGGCAGC-3′. U6 reverse, 5′-GGGTCCGAGGTGCACTGGATACGACAAAATATGG-3′; GAPDH forward, 5′-CTCCCGCTTCGCTCTCTG-3′, GAPDH reverse, 5′- CTGGCGACGCAAAAGAAG-3′, TP53 forward: 5′-GGCCCACTTCACCGTACTAA-3′; TP53 forward: 5′-GTGGTTTCAAGGCCAGATGT-3′.

### Western blot

Whole cell lysates were suspended in 1 × SDS loading buffer, boiled at 95 °C for 5 min, and centrifuged. Supernatants were separated on SDS–10% PAGE and transferred onto PVDF membranes (Bio-Rad, USA). Membranes were blocked in 5% milk in PBST (10 mM phosphate buffer, pH 7.2; 150 mM NaCl; and 0.1% Tween-20) for 60 min, washed three times, and incubated with appropriate antibody at 4 °C overnight. Membranes were washed three times with PBST, incubated with horseradish peroxidase-conjugated secondary antibody at 1:5,000, and developed by Immun-Star HRP Substrate (Bio-Rad, CA, USA). Blots were probed with β-actin antibody (Sigma-Aldrich, USA) as the loading control.

### Dual Luciferase Assay

3′-UTR segment *of TP53* gene corresponding to predicted target site was amplified by PCR from human genomic DNA using primers that included a XbaI and EcoRI tails on the 5′ and 3′ strands, respectively, as previously described^20^. PCR products were restriction digested with both XbaI and EcoRI DNA restriction endonucleases, gel purified, and ligated into pGL3 vector (Promega, USA). HT29 cells were transfected with the firefly luciferase UTR-report vector, control Renilla luciferase pRL-TK vector (Promega, USA) with Lipofectamine 2000 reagent, according to the manufacturer’s protocol (Invitrogen, USA). Twenty-four hours after transfection, cells were lysed with a 1x passive lysis buffer and the activity of both Renilla and firefly luciferases were assayed using the dual-luciferase reporter assay system (Promega, USA), according to the manufacturer’s instructions.

### Cell proliferation Assay

MTT [3-(4, 5-dimethylthiazol-2-yl)-2, 5-diphenyltetrazoliumbr-omide] - based assay was performed to estimate the effect of miR-150-5p mimics, anti-miR-150-5p, or TP53 siRNA on human colonic adenocarcinoma cells’ proliferation, as previously described^21^. Cells were seeded into 96-well plates (5,000 cells/well in 200 µL medium) and incubated for 24 hrs. HT29 cells were transfected with miR-150-5p mimics, anti-miR-150-5p, or TP53 siRNA using Lipofectamine 2000 Reagent (ThermoFisher Scientific, USA) as indicated. Cells cultured in complete medium were used as control. At the end of incubation, 20 µL of 5 mg/ml MTT (Sigma, USA) solution was added per well, and the cells were incubated for another 4 hr at 37 °C. Supernatants were removed and formazan crystals were dissolved in 150 µL of DMSO (Sigma-Aldrich, USA). OD was determined at 490 nm using multi-microplate test system (InfiniteM200Pro,USA).

### Statistical analysis

All results were expressed as mean ± standard deviation. We used Student’s *t*-test or ANONA (One-way) to compare two and three groups, respectively. P < 0.05 was considered significantly different.

## Results

### miR-150-5p directly targets TP53 in CRC

**Figure 1:**
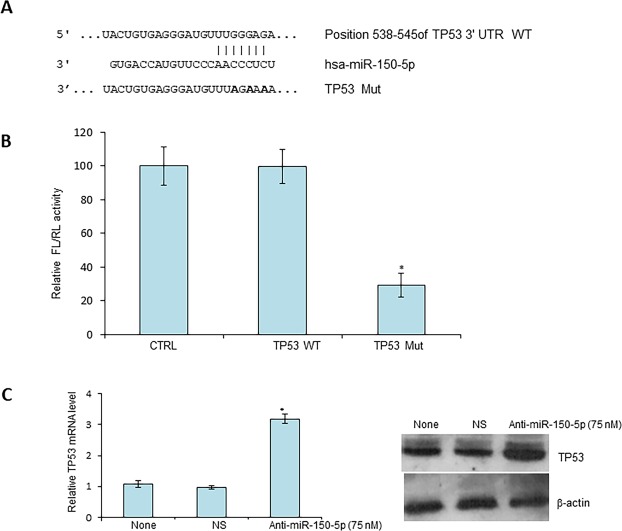
miR-150-5p directly targets TP53 in CRC. (**A**) Predicted target site of miR-150-5p in 3′-UTR region of TP53 mRNA. WT, wild-type, Mut, mutation. (**B**) Equal amounts of empty vector (FL), those with wild-type TP53 3′-UTR or mutant and internal control vector (RL) was transiently transfected into HT29 cells for 48 hrs followed by dual-luciferase assays. (**C**) HT29 cells were transiently transfected with non-specific (NS) miRNA mimics or anti miR-150-5p (50 nM) for 48 hr and mRNA (left panel) and protein levels (right panel) were determined by Western blot and RT-PCR, respectively. *P < 0.01 (N = 3).

TP53 is a tumor suppressor gene as well as putative target of miR-150-5p^18^. To investigate whether miR-150-5p targets TP53 in CRC, we first performed bioinformatic analysis, using TargetScan and found that there are multiple miR-150-5p target sites in TP53 mRNA (Fig. 1A).

**Figure 2:**
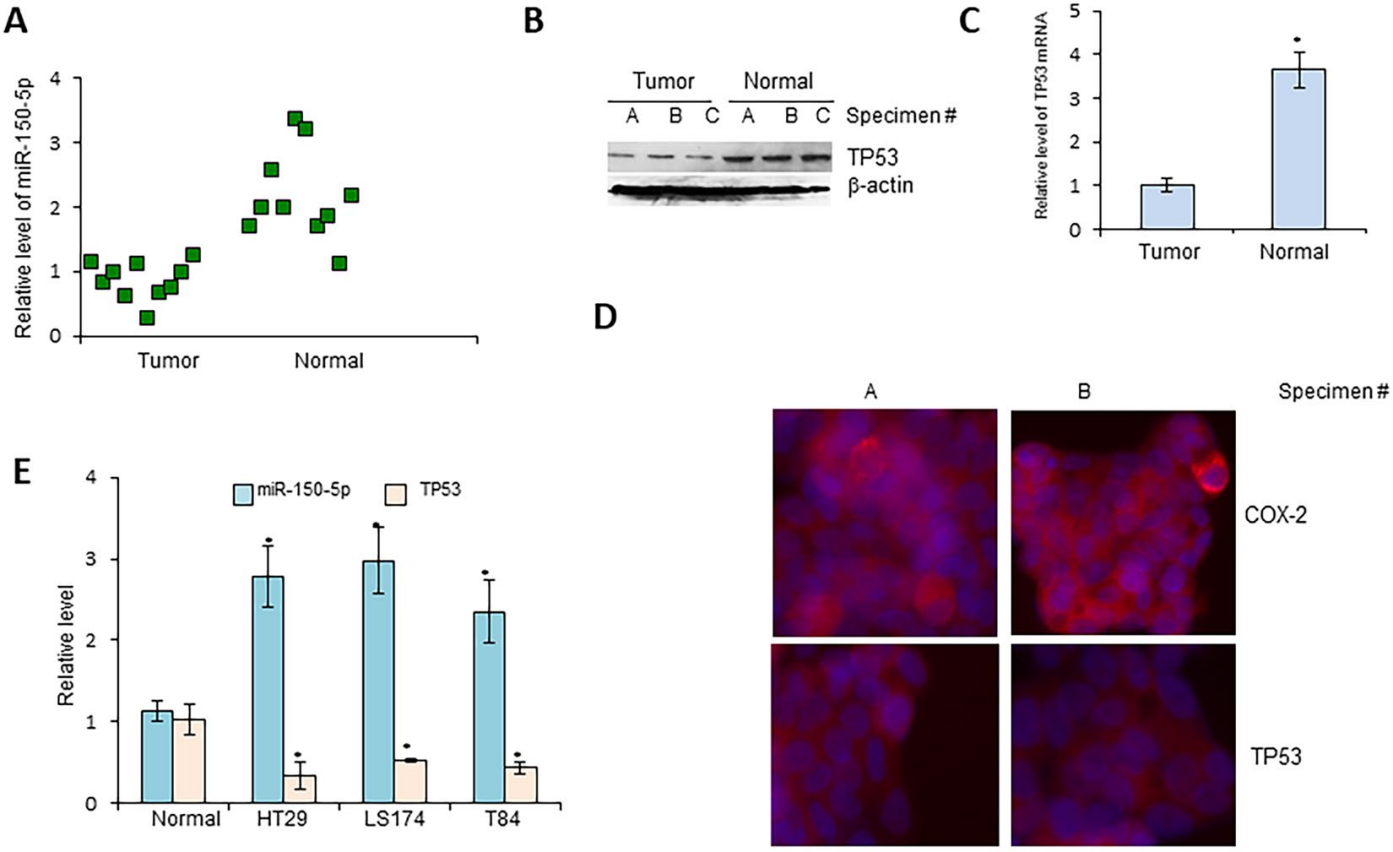
Expression of miR-150-5p and TP53 in colon cancer specimens and cell lines. The expression levels of miR-150-5p (**A**) or TP53 protein (**B**) or TP53 mRNA (**C**) were determined in both colon adenocarcinoma (tumor) and non-cancerous (normal) adjacent tissues. (**D**) Representative images of immunofluorescence staining, using antibodies against TP53 or COX-2, on isolated cancer cells purified from 3 select specimens. (**E**) Expression levels of miR-150-5p and TP53 mRNA, as determined by RT-qPCR, in colon adenocarcinoma cell lines (N = 3) and the normal human colon epithelial cell line CCD 841 CoN. *P < 0.01.

To validate a direct interaction between miR-150-5p and TP53, we cloned a miR-150-5p target site at 3′-UTR of TP53 mRNA upstream of firefly luciferase (FL) reporter gene, so that it could be regulated by miR-150-5p. In addition, a mutated version of this target site was used as the negative control. Colon adenocarcinoma HT29 cells were transfected with either the control FL plasmid or FL plasmid with miR-150-5p target site or the FL plasmid with mutation of miR-150-5p target site. Also, *Renilla* luciferase reporter plasmid was co-transfected as an internal reference. As shown in Fig. 1B, a significant decrease in FL activity was observed in cells transfected with FL reporter with wild type of miR-150-5p target site. In contrast, no repression of FL activity was obtained in cells transfected with miR-150-5p-mutation FL reporter plasmid (Fig. 2B). Further, we confirmed a direct targeting of TP53 mRNA by miR-150-5p in HT29 cells. We found that TP53 increased at both protein and mRNA levels when cells were treated with miR-150-5p specific inhibitors (Fig. 1C). Taken together, these results indicate that TP53 is a direct target of miR-150-5p in CRC cells.

### Coupled increase of miR-150-5p and decrease of TP53 in CRC

Next, we sought to determine miR-150-5p and TP53 levels in in tissue specimens derived from CRC patients. Total RNAs were extracted from 10 colon cancer and adjacent non-cancerous tissue samples for the assessment of miR-150-5p and TP53 mRNA levels by quantitative RT-PCR. TP53 protein levels were assessed by Western blots as well. In the meantime, the same tissues were subjected to purification of colon cancer cells followed by immunofluorescence staining (IF). We found that miR-150-5p was significantly up-regulated in CRC cancer tissues (Fig. 2A). In contrast, TP53 mRNA and protein levels decreased by an average of >45% (Fig. 2B,C), compared to the non-cancerous adjacent colon mucosa. In addition, weak cytoplasmic staining for TP53 was observed in the cancerous tissues, in contrast to strongly positive staining of COX-2, another biomarker for CRC^22^ (Fig. 2D).

Next, we used RT-PCR to determine levels of miR-150-5p and TP53 in different colon adenocarcinoma cell lines and similar results were obtained in all three colon adenocarcinoma cell lines as found in the primary tissues (Fig. 2E). Collectively, these results suggest that miR-150-5p and TP53 are negatively correlated in CRC, suggesting a possible regulation of TP53 by miR-150-5p.

### miR-150-5p stimulates cell proliferation *in vitro*

**Figure 3:**
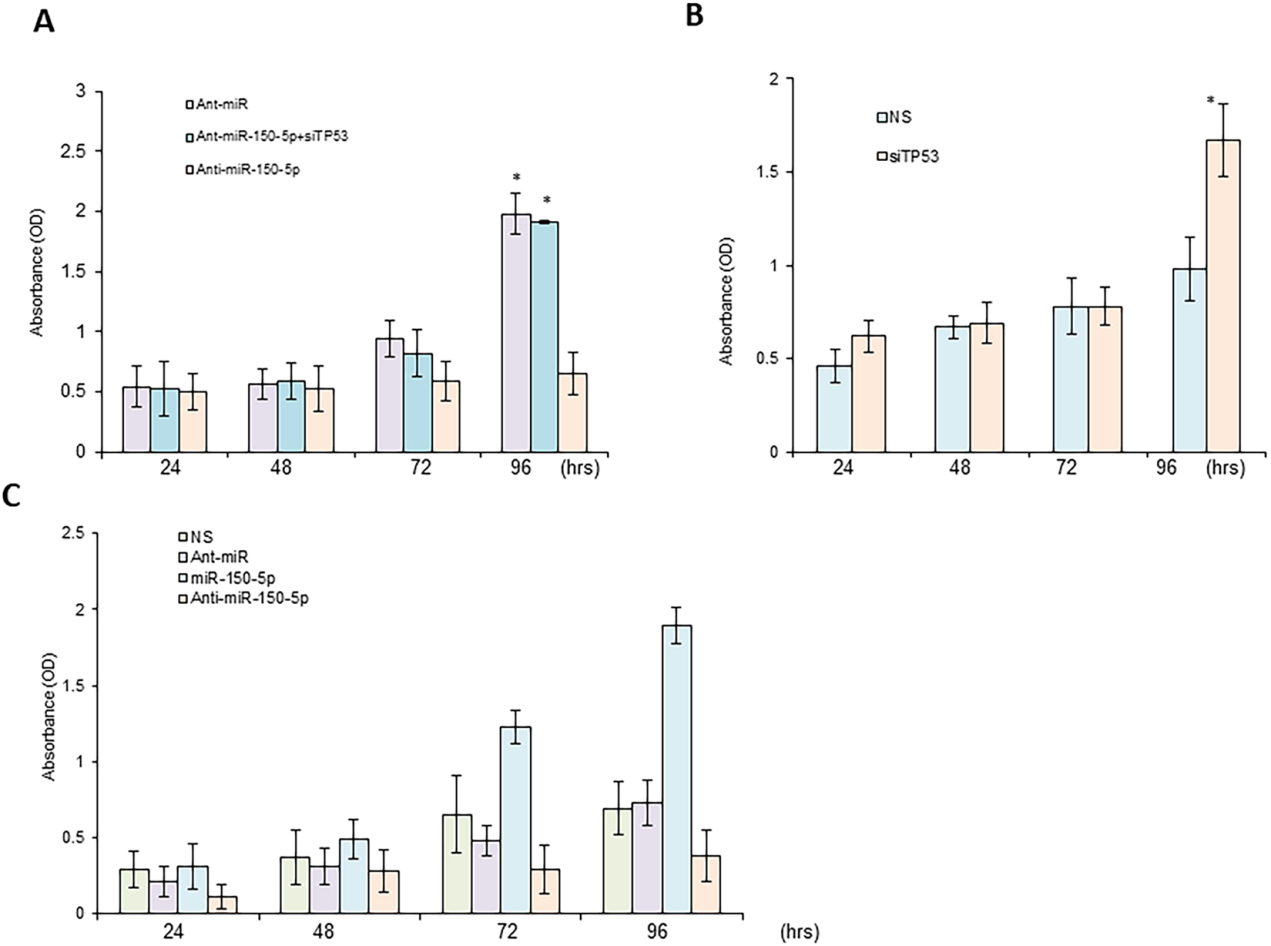
Effects of miR-150-5p-TP53 signaling on proliferation of CRC *in vitro*. (**A**) HT29 cells were transiently transfected with non-specific miRNA mimics (NS) or antagomirs (anti-miR), or miR-150-5p mimics or anti-miR-150-5p for the time period as indicated. (**B**) HT29 cells were transiently transfected with non-specific siRNA (NS) or TP53 siRNA (siTP53) for time period, as indicated. (**C**) HT29 cells were transfected with non-specific antagomirs (NS), or anti-miR-150-5p, or anti-miR-150-5p combined with TP53 siRNA as indicated. Cell proliferation was analyzed by MTT assay. *P < 0.01 (N = 3).

HT29 cells were transfected with non-specific miRNA mimics, anti-miR-150-5p, or anti-miR-150-5p combined with TP53 siRNAs, as indicated, and MTT assays were performed to determine cell proliferation. As shown here, miR-150-5p mimics promoted, but miR-150-5p inhibitors inhibited cancer cell growth (Fig. 3A). Consistent with this, depletion of TP53 also promoted cancer growth, and abolished anti-miR-150-5p mediated inhibition of cell proliferation (Fig. 3B,C). These results further confirm that miR-150-5p directly inhibits TP53 to exert oncogene functions and promote CRC cancer cell proliferation.

### Inhibition of miR-150-5p affects the expression of key cell cycle factors

**Figure 4:**
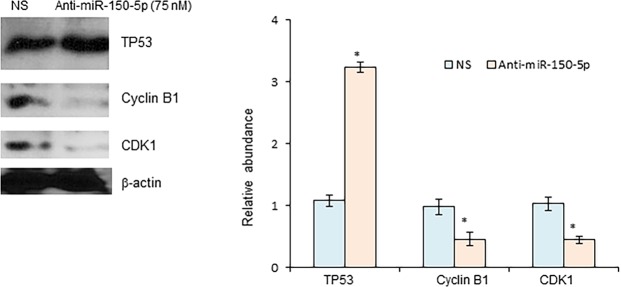
Effects of miR-150-5p on cell cycle in CRC. HT29 cells were transiently transfected with anti-miR-150-5p. The expression levels of cell cycle regulators cyclin B1 and CDK1 were determined and quantified by Western blot. *P < 0.01 (N = 3).

TP53 has been found to induce cell cycle arrest in G2/M phase in clear cell renal cell carcinoma (ccCRC)^23^. Here we tested the role of miR-150-5p-TP53 signaling in regulation of cell cycle in CRC. The expression of cell cycle regulators associated with G2/M phase transformation was investigated in HT29 cells treated with either anti-miR-150-5p or the control non-specific miRNA mimics. As shown in Fig. 4, protein levels of cyclinB1 and CDK1 were significantly decreased in cells transfected with anti-miR-150-5p, in contrast to upregulation of TP53. These results support that miR-150-5p-TP53 signaling pathway modulates cell cycle progression via expression of cell cycle regulators.

### TP53 triggers cell apoptosis in CRC cells

**Figure 5:**
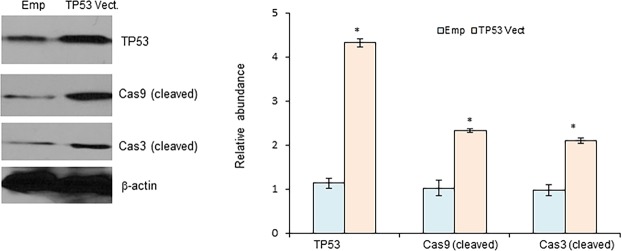
TP53 induces cell apoptosis in CRC cells. HT29 cells were transiently transfected with empty vector, pcDNA3.1 (Emp) or pcDNA3.1 with open reading frame of TP53 (TP53 vect., 1 μg) for 48 hrs. The expression levels of cleaved caspase-3 and caspase-9 were determined and quantified by Western blot. *P < 0.01 (N = 3).

Evasion of apoptosis is a critical event during malignant transformation, and TP53 has been found to induce apoptosis in various cancer cells^24^. Hence, we examined the effects of TP53 on induction of CRC cell apoptosis. As shown in Fig. 5, the expression levels of cleaved caspase-3 and caspase-9, which are molecular hallmarks for cell apoptosis, were significantly increased in HT29 cells transfected with pc-DNA3-TP53. Collectively, these results suggest that TP53 overexpression exerts significant effects on the proliferation of CRC cells.

## Discussion

The molecular mechanisms underlying CRC initiation and progression remain largely unknown, and, therefore, a comprehensive detailing of all such mechanisms is critical. This alone will facilitate discovery of novel biomarkers for early diagnosis and therapy, thus improving the outcome of CRC patients. Over the past decade, miRNAs have emerged as a new and promising class of gene regulators involved in cancer progression^25,26^. Here, we show, for the first time, that miR-150-5p plays a critical role in colon tumorigenesis by inhibiting the tumor suppressor gene TP53 to promote cancer proliferation. Our results suggest that miR-150-5p can be used for early stage diagnosis and a therapeutic target for therapy of CRC. These collective findings show an important and new role of miR-150-5p-TP53 signaling pathway in carcinogenesis of CRC, and provide a molecular detaining for the correlation between elevation of miR-150-5p and cell proliferation in CRC. Therefore, our study offers a concrete base for further understanding of the complex biology involved in CRC cancer progression.

miRNAs are well-known to play a role in cancer progression. As demonstrated previously by several groups, the same miRNA might play a distinct role in different types of cancer. For example, miR-146a acts as oncogenic as well as tumor suppressive in distinct cell types^27,28^. Consistent with this observation, patients with higher miR-150-5p levels usually have a worse survival in melanoma and inhibition of miR-150-5p enhanced cell apoptosis via upregulation of PDCD4-mediated activation of caspase-8 and p21^29^. However, another study has shown that miR-150 might serve as a potential therapeutic sensitizer through inhibition of the AKT pathway in NK/T cell lymphoma treatment^30^. Therefore, more studies are needed to elucidate the role of miR-150-5p in other types of cancer.

Our results are consistent with the earlier published literature that TP53 plays a protective role in blocking a decisive step in oncogenesis. Activation of TP53 can induce both the mitochondrial and the death-receptor-induced apoptotic pathways^31^. Activation of TP53 leads to expression of pro-apoptotic Bcl-2 (B-cell lymphoma-2) family proteins, mainly Bax, Noxa and PUMA, but inhibits the pro-survival Bcl-2, and in turn, permeabilization of outer mitochondrial membrane. Then cytochrome c releases from the mitochondria binds to APAF-1, and induces the activation of the initiator caspase-9, eventually resulting in the activation of executioner caspase-3, -6 and -7. On the other hand, activated TP53 increases some death receptors and form death-inducing signaling complex with caspase-8, to induce apoptosis. The progression of cell cycle is tightly controlled by cyclins and cyclin-dependent kinases (CDK). p21 is one member of CDK inhibitor family, which stimulates cell cycle transition from G1 to S phase, as well as one of the major mediator of p53-induced growth arrest. In response to DNA damage, p53 induces not only cell cycle G1 phase arrest, but also G2/M checkpoint arrest^32^.

In conclusion, our results support a role of miR-150-5p-TP53 signaling pathway in the proliferation, cell cycle progress, cell apoptosis and invasion/migration of CRC cells. Furthermore, miR-150-5p acts an oncogene and its target TP53 acts as a tumor suppressor in CRC pathogenesis. Due to current lack of appropriate biomarkers and therapeutic targets in CRC, these results suggest that miR-150-5p-TP53 signaling may provide novel insights into the improvement of clinical therapeutic strategies for CRC patients.

## Data Availability

All the data is available within this manuscript.
